# Supplementary figures and images for: Extensive genetic differentiation between recently evolved sympatric Arctic charr morphs
Source: Ecol Evol. 2019 Sep 12;9(19):10964–83. doi: 10.1002/ece3.5516 (PMC6802010; doi:10.1002/ece3.5516)

A)

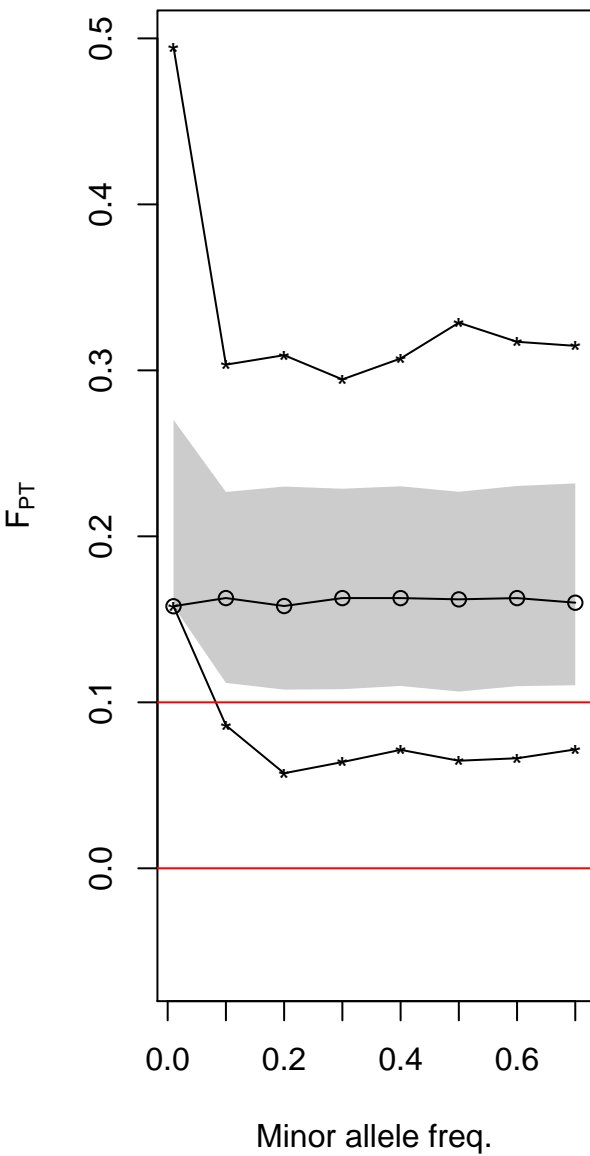

B)

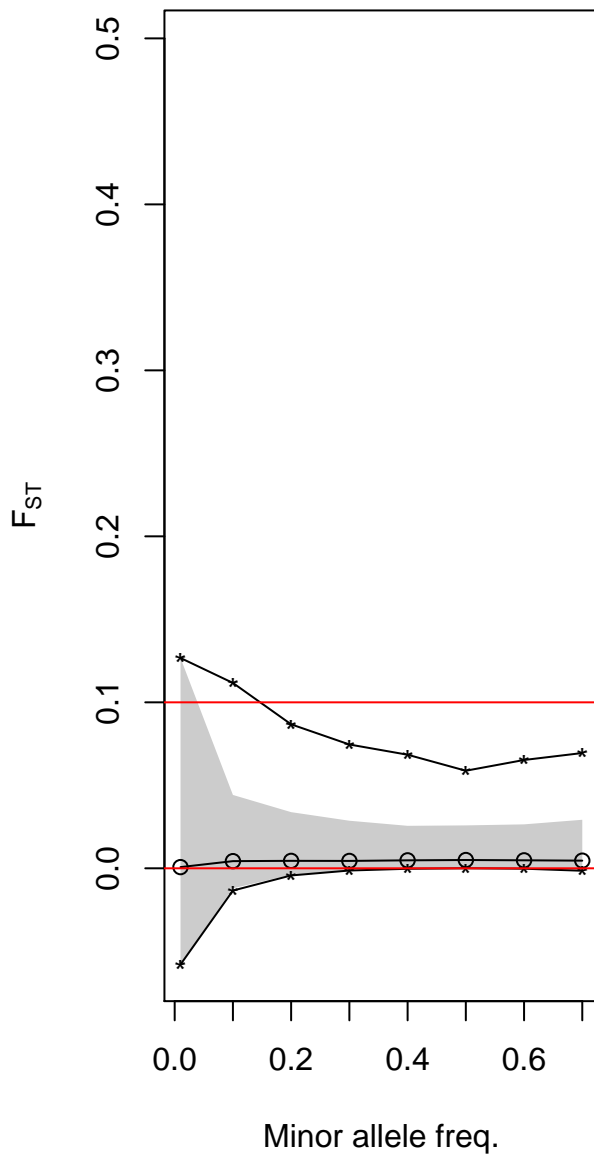

Supplement: Supplementary file 1 [file ECE3-9-10964-s001.pdf]

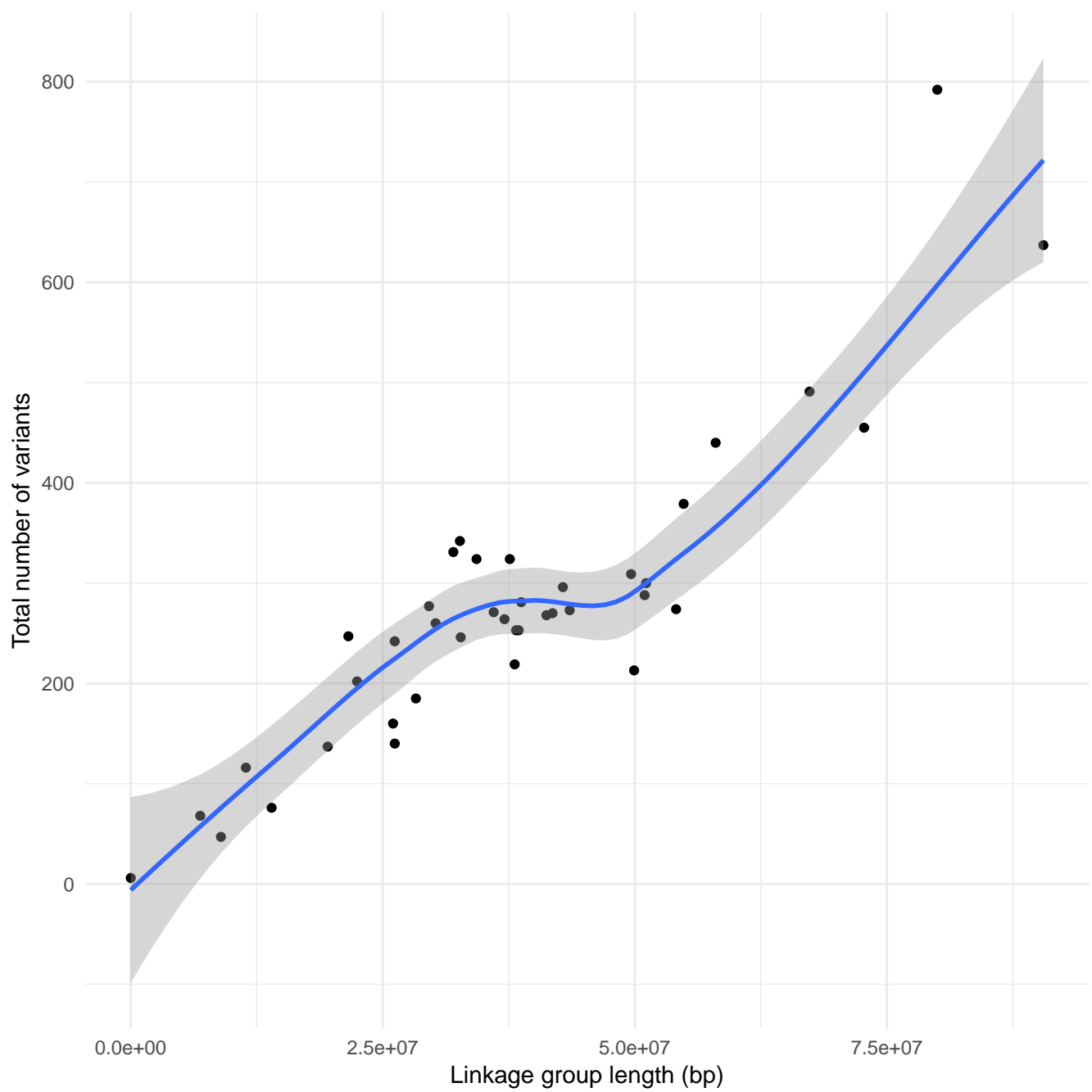

Supplement: Supplementary file 2 [file ECE3-9-10964-s002.pdf]

A)

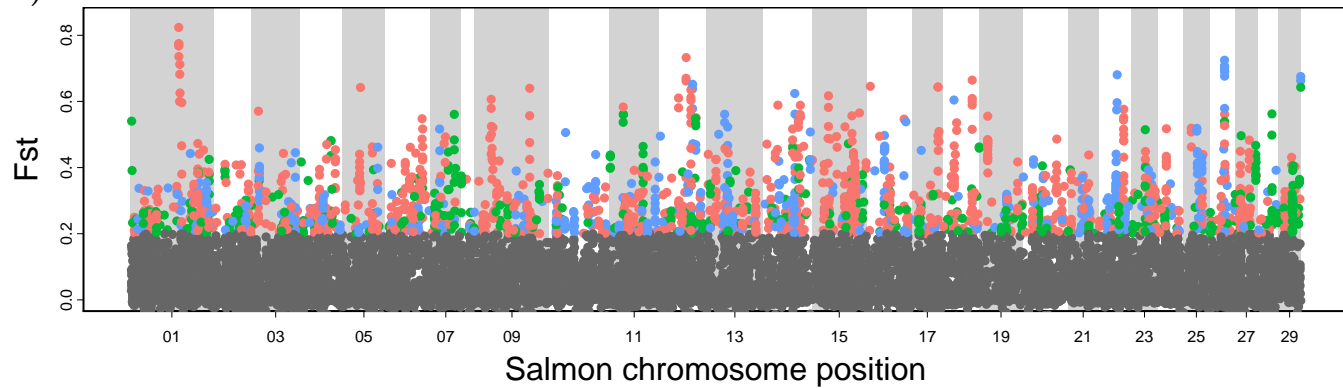

B)

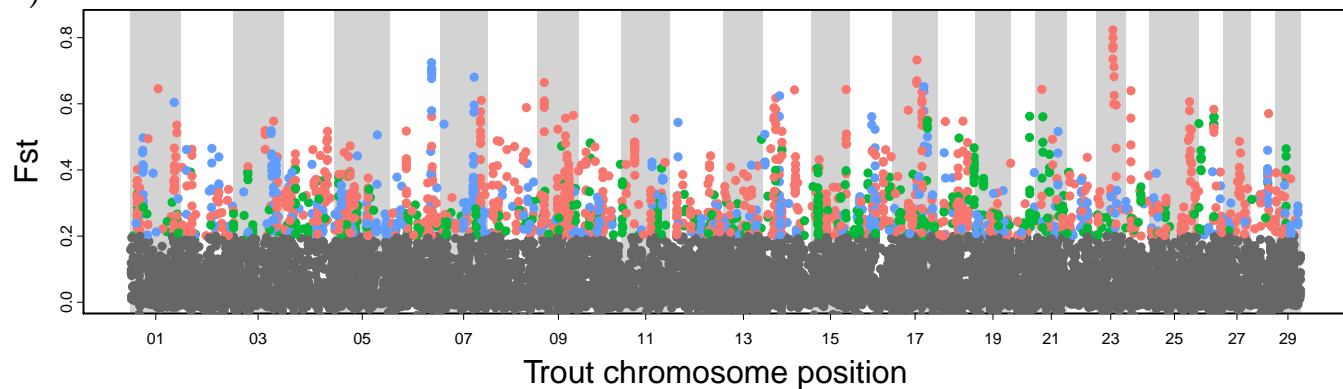

Supplement: Supplementary file 3 [file ECE3-9-10964-s003.pdf]

A)

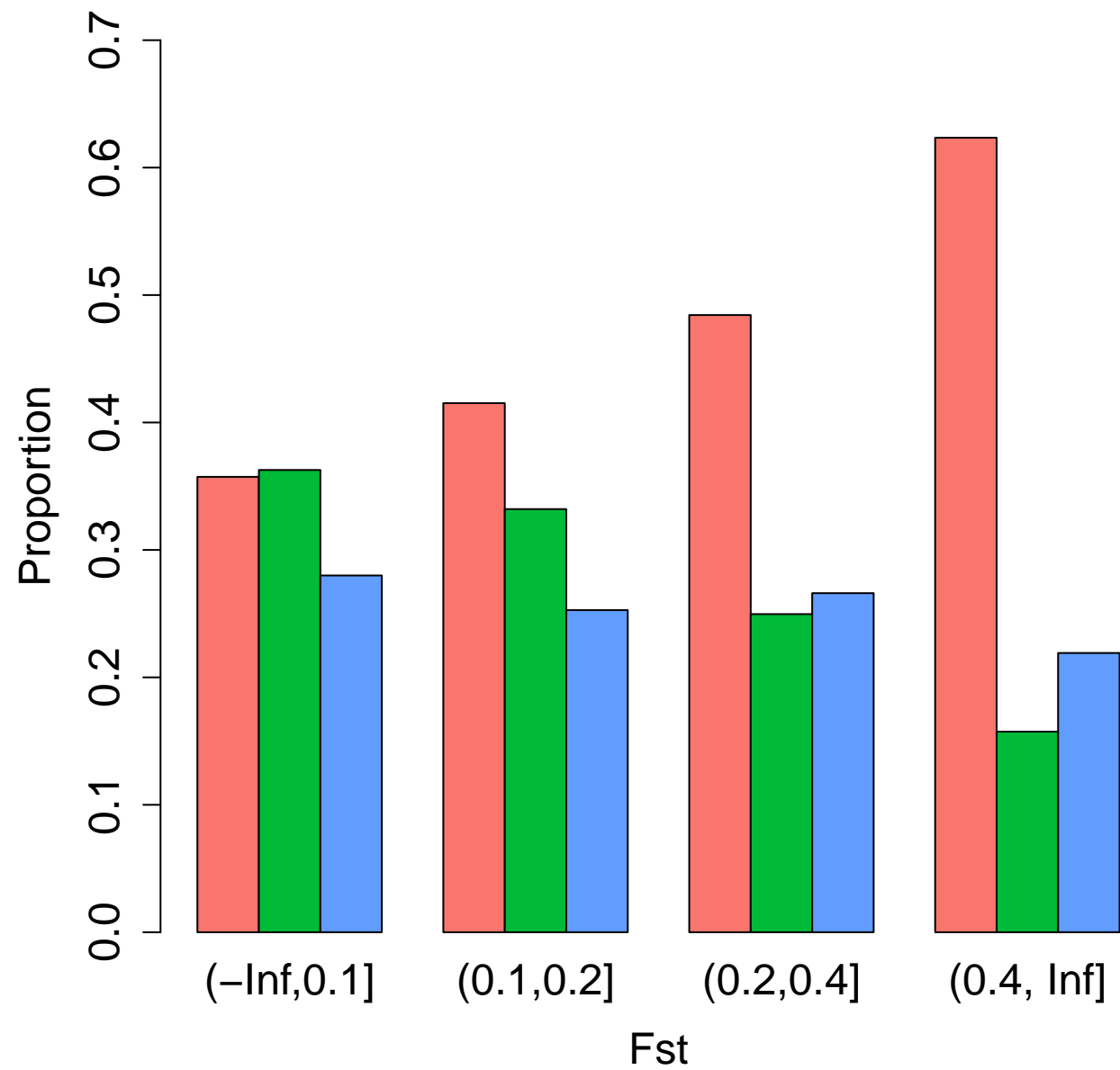

B)

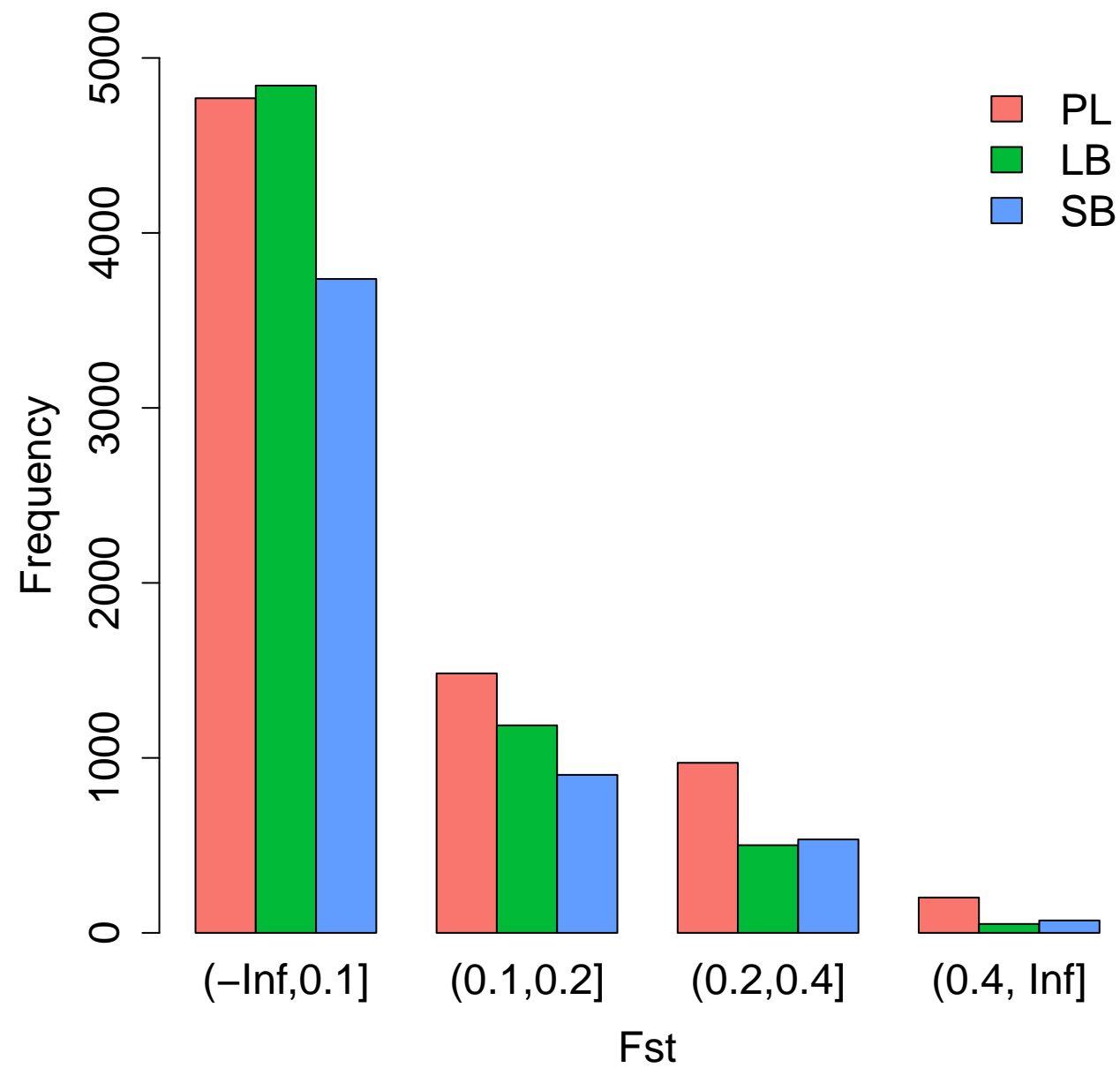

Supplement: Supplementary file 4 [file ECE3-9-10964-s004.pdf]

A)

### Allele frequency

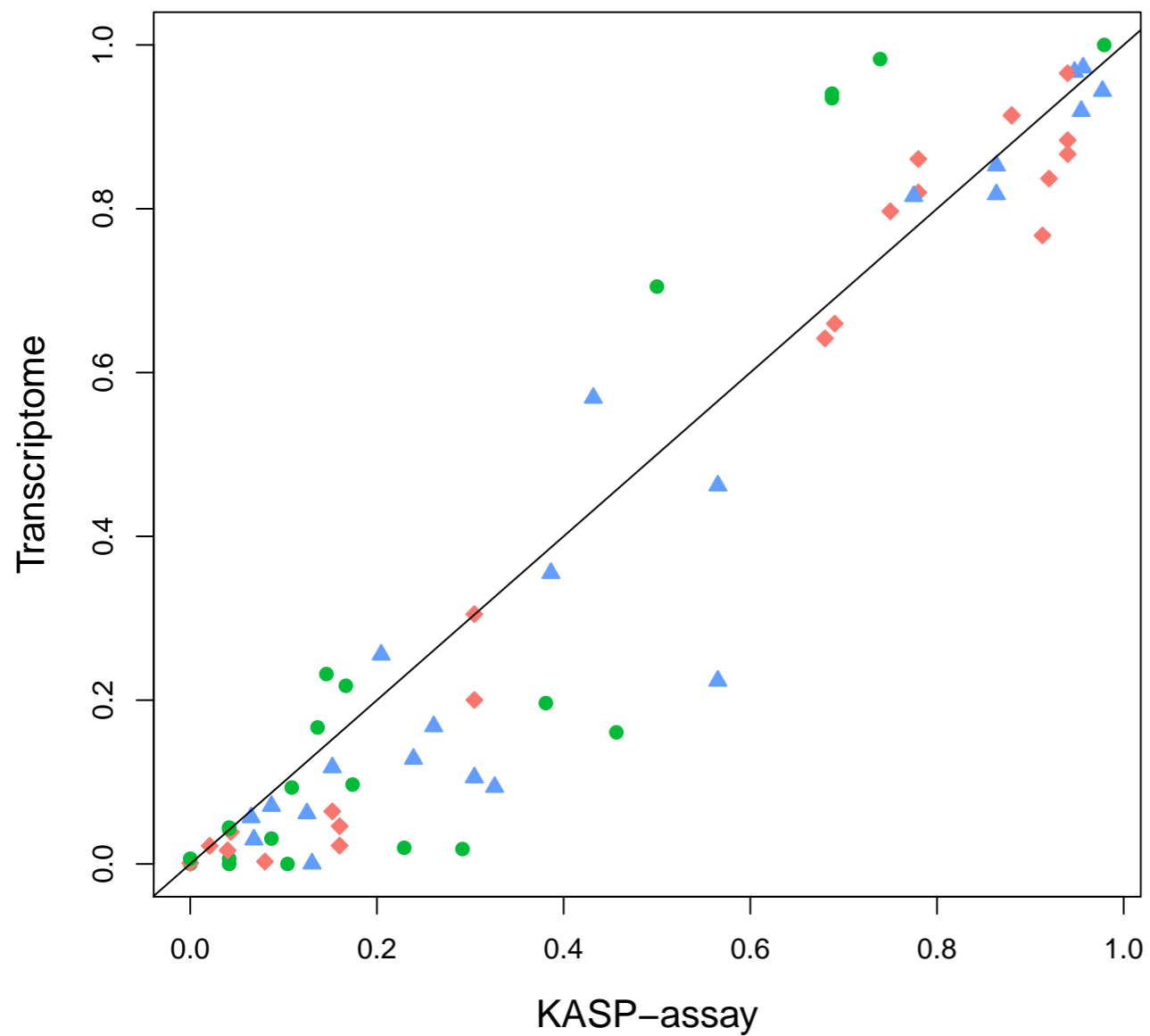

B)

### Fst-values

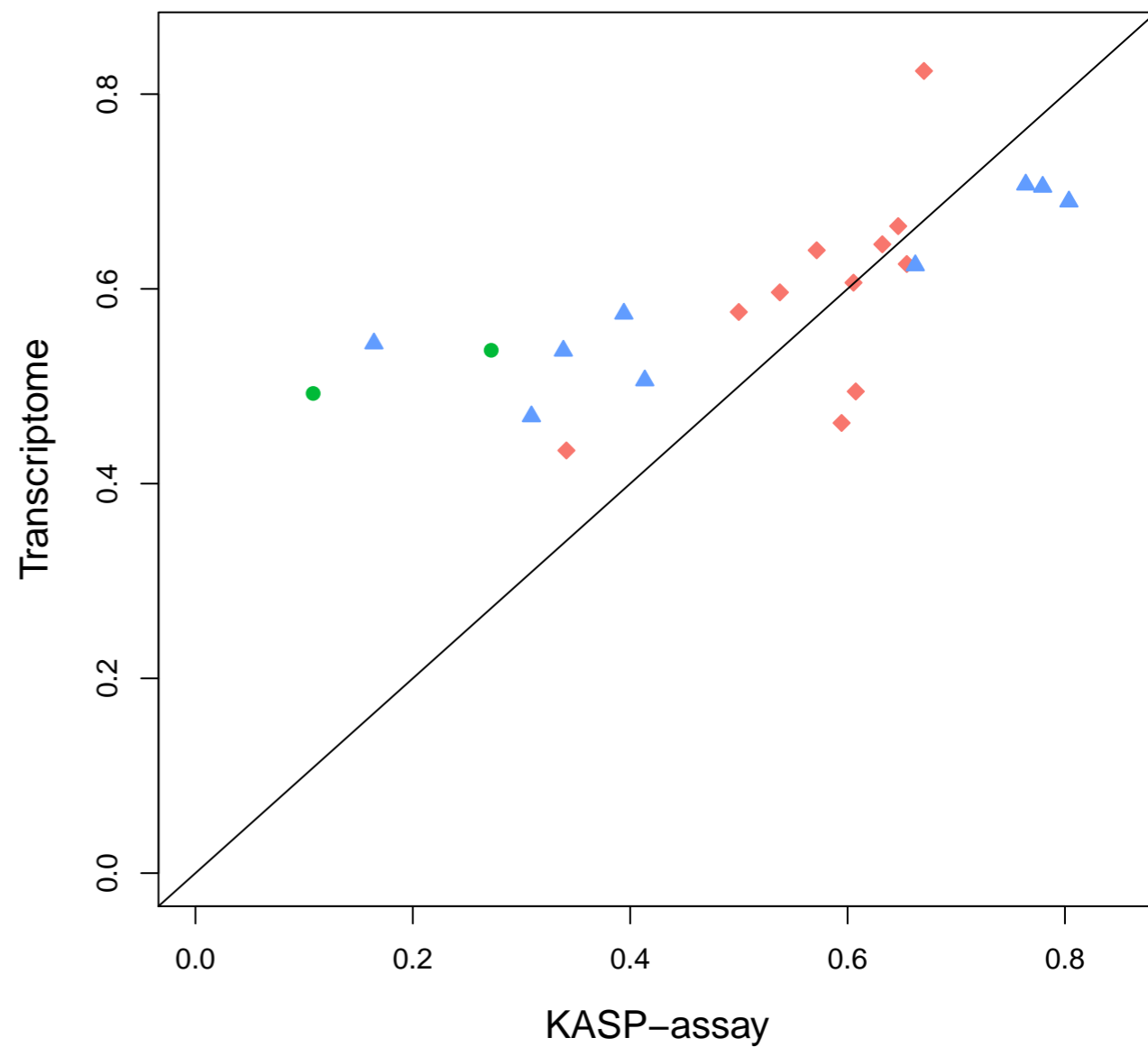

Supplement: Supplementary file 5 [file ECE3-9-10964-s005.pdf]

A)

**calm1**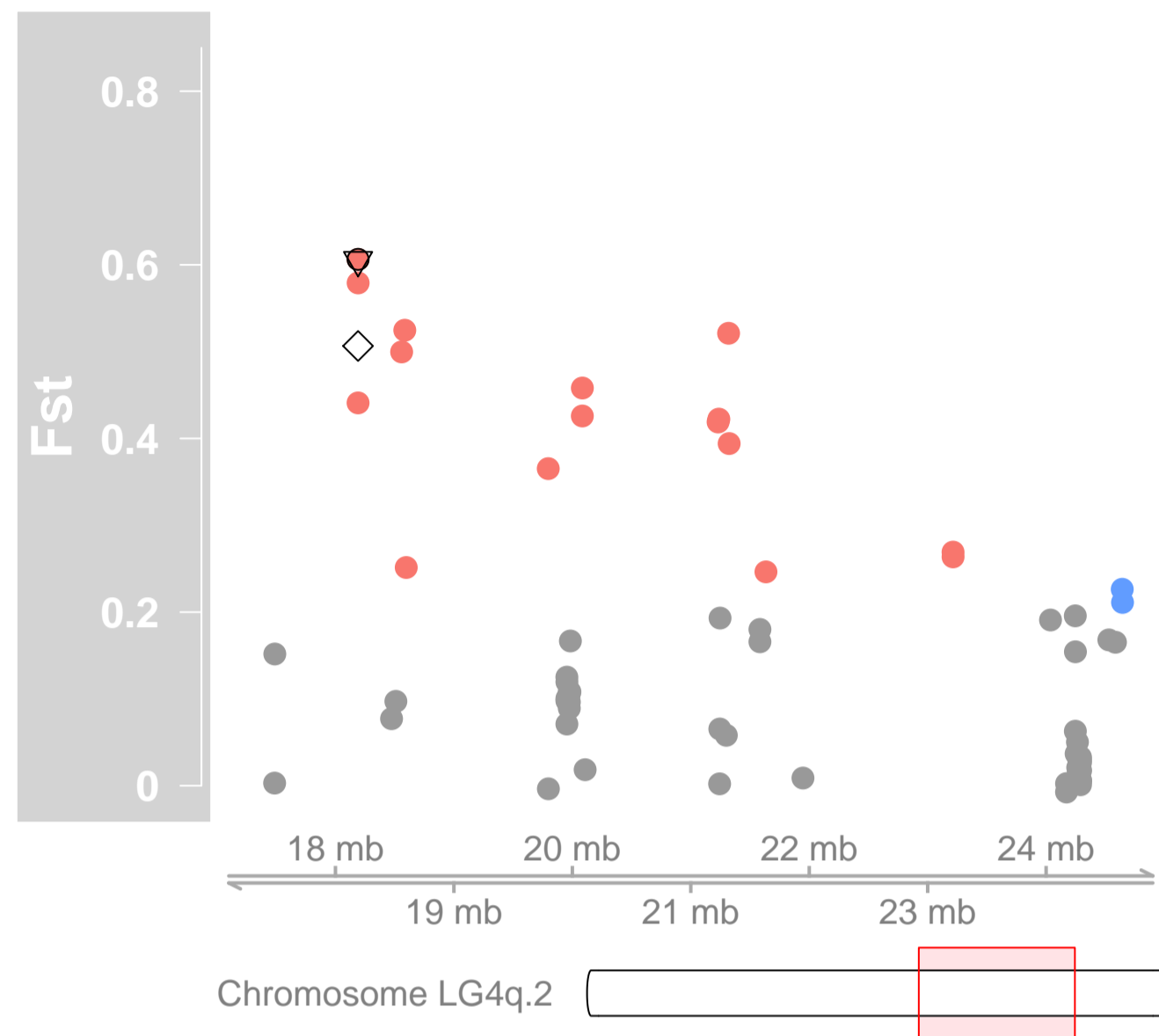

B)

**Kiaa1324, eif4g2b**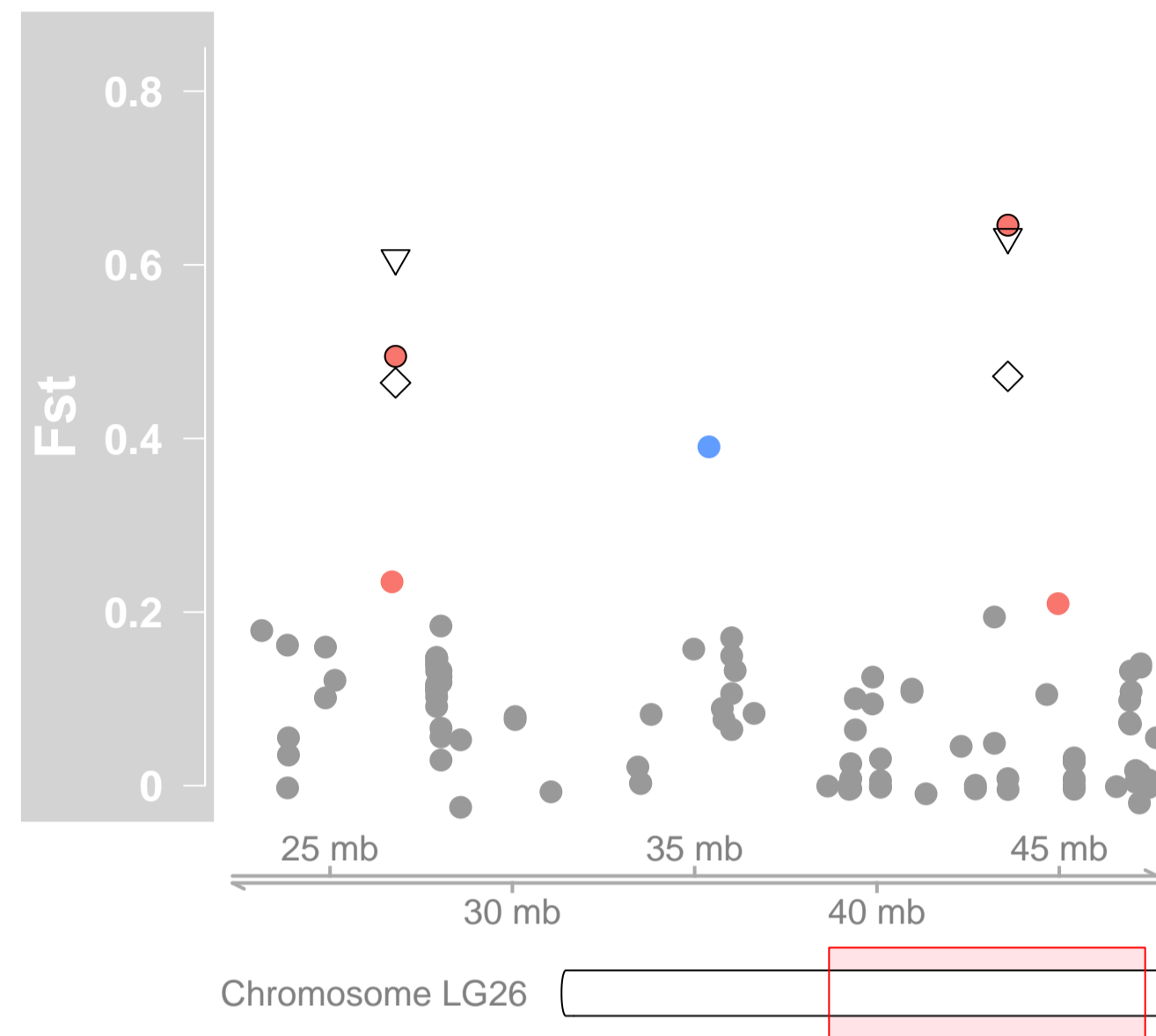

C)

**msi1, tcf15**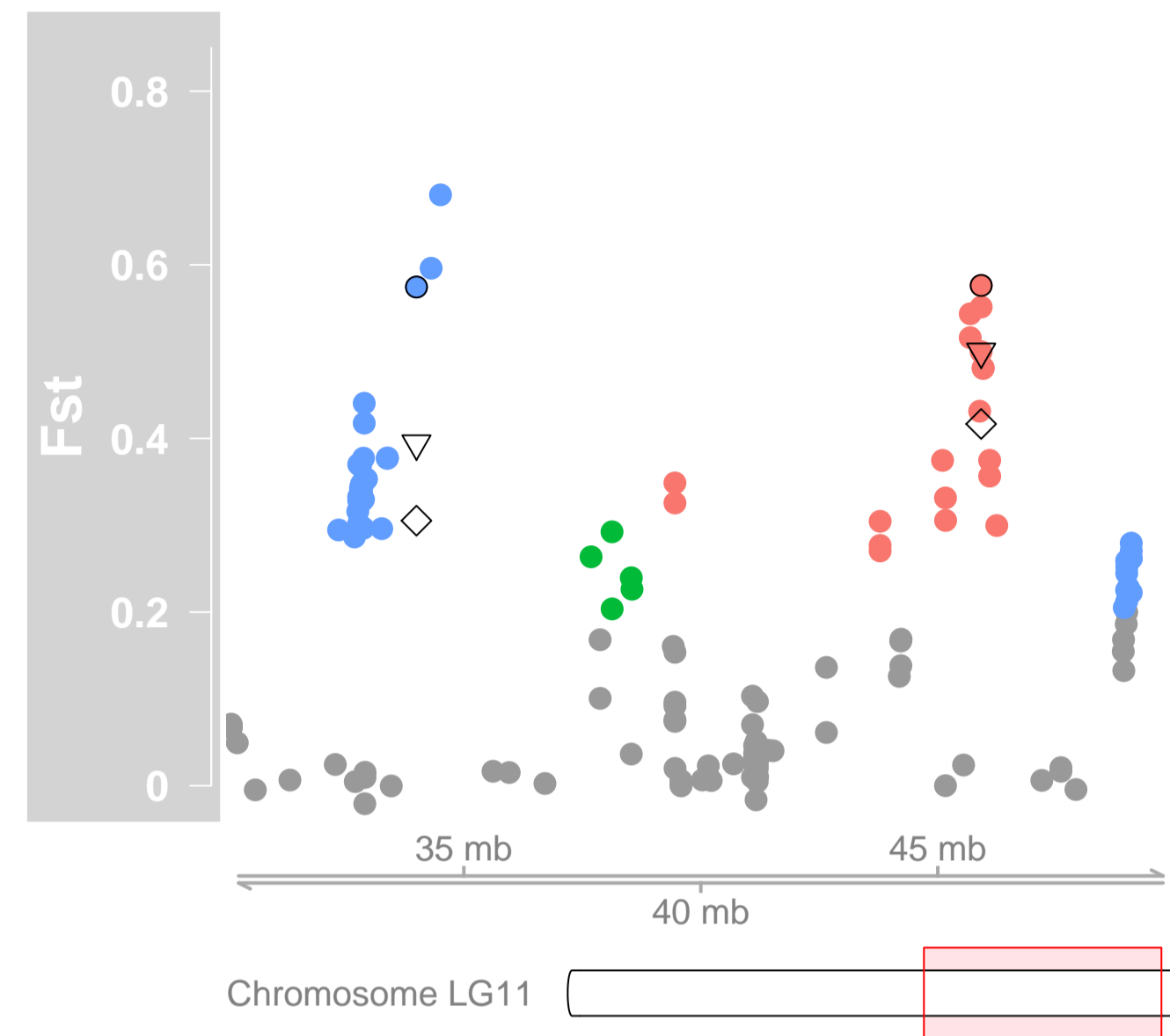

D)

**lrrc1, cox11, adk**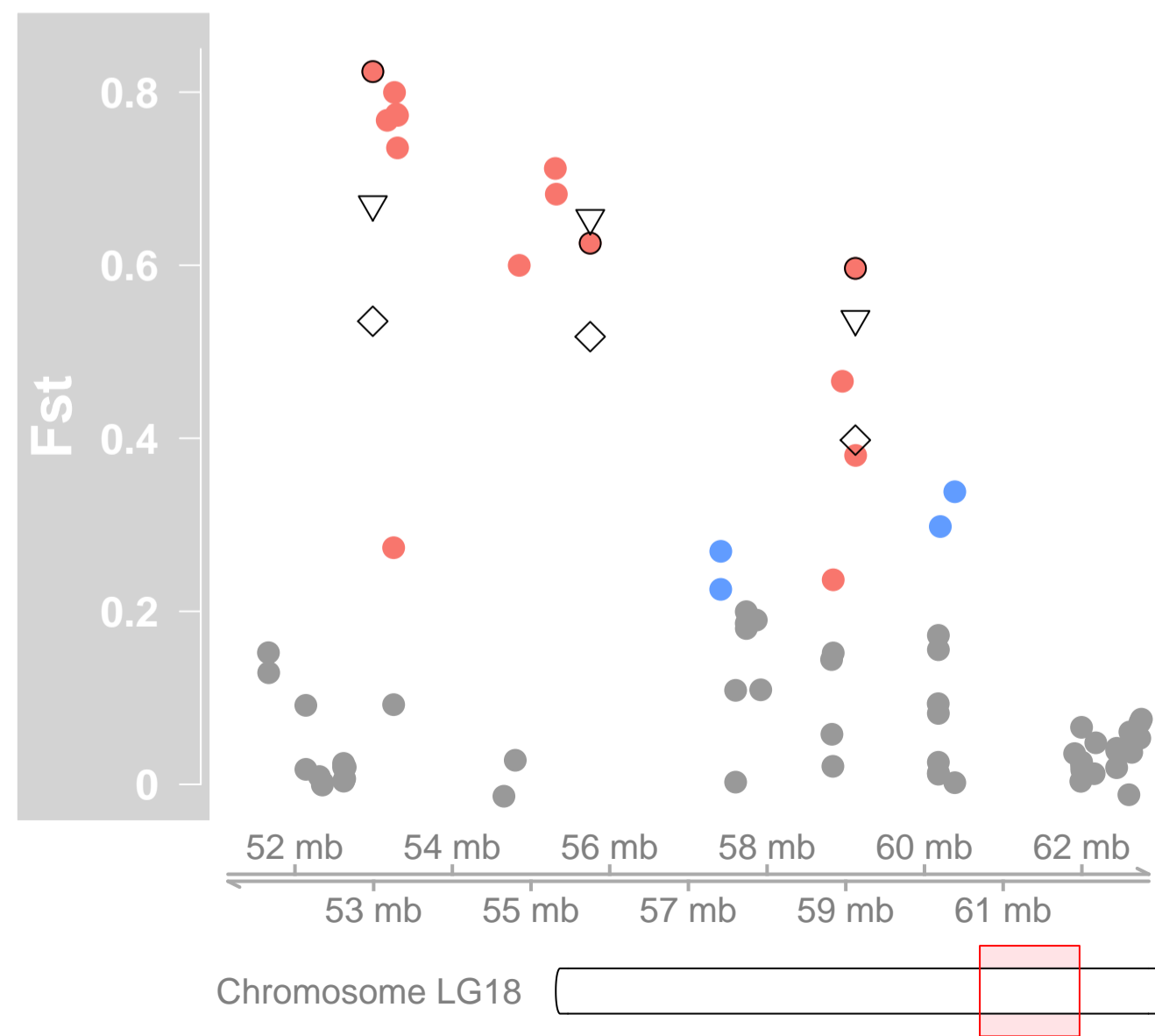

E)

**dennd5a, wee1, gas1l**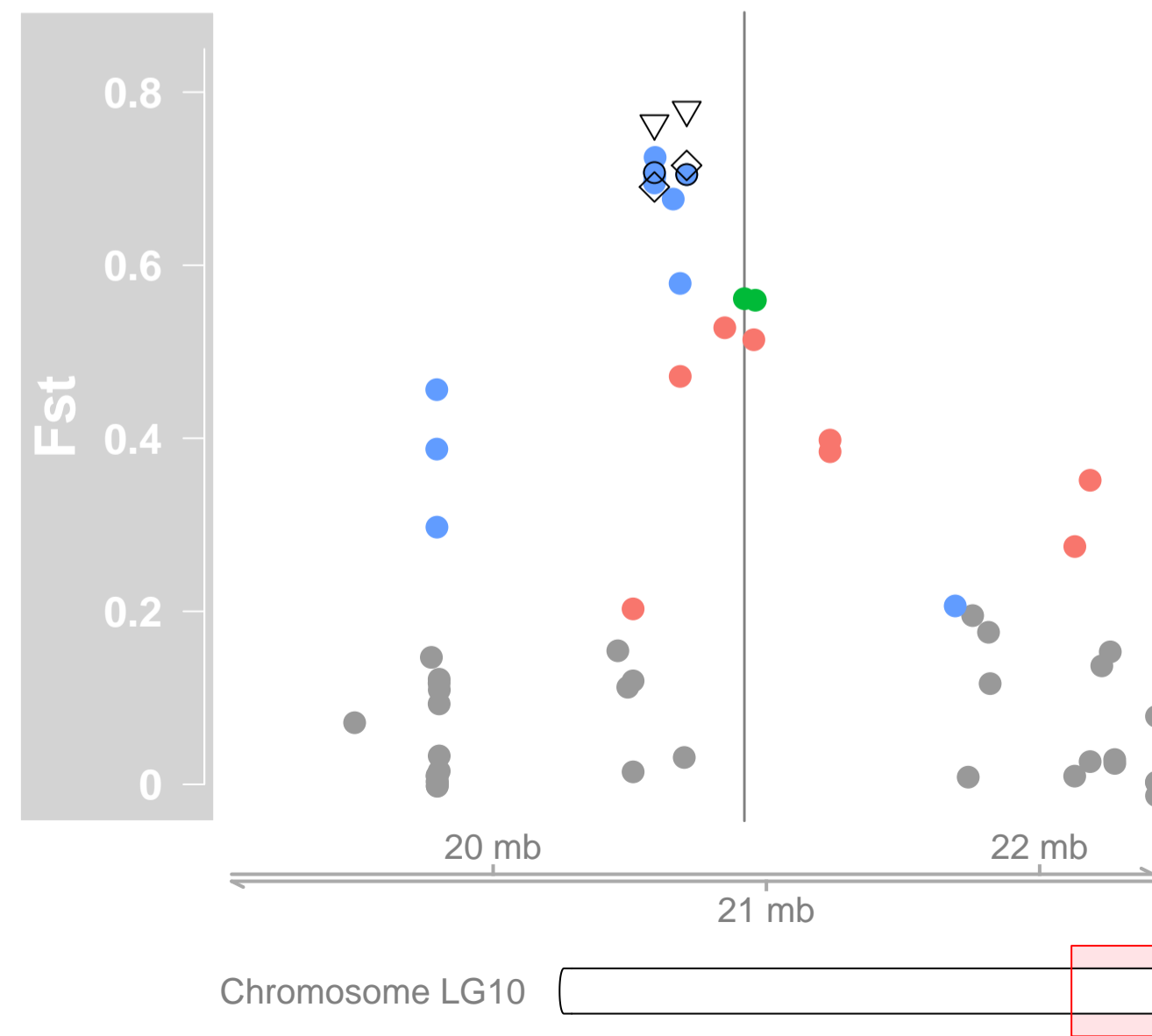

F)

**tmem9b, gas1l**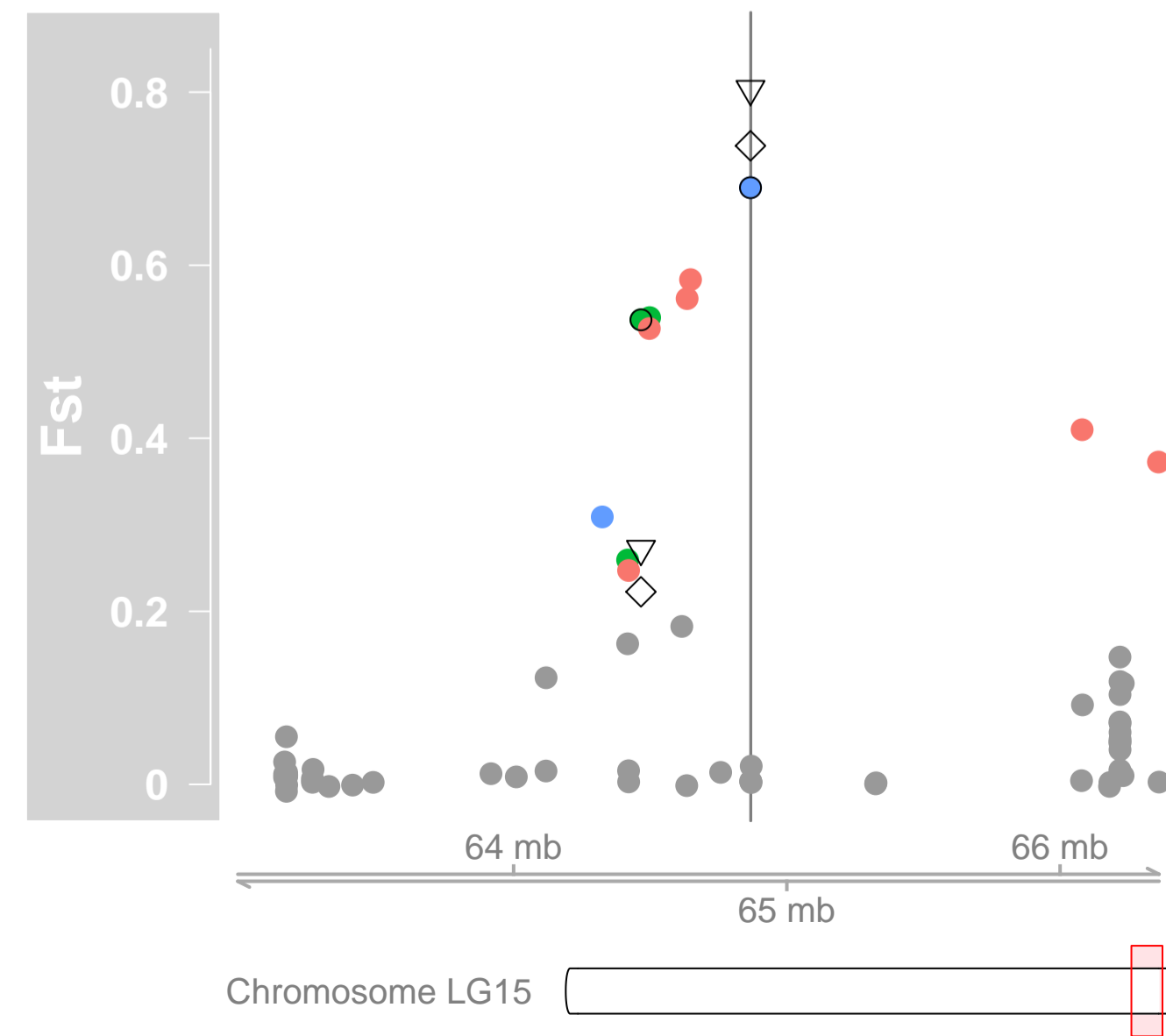

Supplement: Supplementary file 6 [file ECE3-9-10964-s006.pdf]
