# Supplementary material for: Extensive genetic differentiation between recently evolved sympatric Arctic charr morphs
Source: Ecol Evol. 2019 Sep 12;9(19):10964–83. doi: 10.1002/ece3.5516 (PMC6802010; doi:10.1002/ece3.5516)
Supplement: Supplementary file 7 [file ECE3-9-10964-s007.pdf]

Supplementary Material for Extensive genetic differentiation between recently evolved sympatric Arctic charr morphs

Jóhannes Guðbrandsson[1,2]*, Kalina H. Kapralova[1], Sigríður R. Franzdóttir[1,3], Þóra Margrét Bergsveinsdóttir[1], Völundur Hafstað[1], Zophonías O. Jónsson[1,3], Sigurður S. Snorrason[1] and Arnar Pálsson[1,3]

[1] Institute of Life and Environmental Sciences, University of Iceland, 101 Reykjavik, Iceland
[2] Marine and Freshwater Research Institute, 101 Reykjavik, Iceland
[3] Biomedical Center, University of Iceland, 101 Reykjavik, Iceland

Supplementary Material

Supplementary Tables and Files

Table S1: Tab-delimited text file with the position, alleles, alternative frequencies within morphs, F-statistics between morphs and samples and predicted effect of the variant on protein composition for all the variants after the final filtering step.

On figshare: doi:10.6084/m9.figshare.8705888

id: Identifier for each variant from 1 to 19,252
transcript_id: Name of the transcript from the Trinity assembly.
start: Start position of polymorphism within contig.
end: End position of polymorphism within contig.
ref: Base(s) of the reference allele.
alt: Base(s) of the alternative allele.
var_pos: Position of polymorphism within contig.
NCBI_id: Sequence identifier for chromosome or scaffold in genome.
Chr: Name of chromosome.
Chr_pos: Position mapped to in chromosome.
Freq_LB: Frequency of alternative allele within the LB morph.
Freq_PL: Frequency of alternative allele within the PL morph.
Freq_SB: Frequency of alternative allele within the SB morph.
Fst: Estimate of F_{ST} between morphs.
Fpt: Estimate of F_{PT} or variation among samples (pools). See methods for further explanation.
effect: Which effect does the mutation have on the gene, 3'- and 5' UTR indicate mutations in those regions, synonymous do not change the reading frame but mutations that do so are coded as transitions from one amino acid to another (single letter a.a. code)
gene_name: Gene name based on SalmonBase annotation.

Table S2: VCF-file with the final set of variants after all filtering steps.

On figshare: doi:10.6084/m9.figshare.8718695

*Corresponding author – Tel: +354-575-2615 – johannes.gudbrandsson@hafogvatn.is

Table S3: Variants only found within one morph. Position within contig and genome, alleles, frequency within the morph, F_{ST}-values and predicted effect are shown.

Transcript id	Var pos	Ref	Alt	Chr	Chr pos	Freq	F_{ST}	F_{PT}	Effect	Gene name
**Private to LB**										
T143531c2g1i1	545	G	A	LG2	1,668,110	0.33	0.19	0.32		CIGSSA_083607.t3
T147645c2g1i1	571	T	A	LG18	49,590,368	0.34	0.24	0.39		AN1-type zinc finger protein 4-like, isoform X1
T149060c0g1i1	83	T	A	LG4q.1:29	13,785,703	0.46	0.16	0.41	UTR	Prenylcysteine oxidase
T149651c0g1i1	420	C	G	LG18	47,847,696	0.34	0.21	0.43	UTR	40S ribosomal protein S24, isoform X3
T161481c0g1i2	203	C	T	LG13	50,967,669	0.45	0.30	0.68	UTR	
T161641c2g2i1	1119	G	A	LG23	40,674,682	0.37	0.24	0.34	UTR	Ubiquitin-conjugating enzyme E2 D2|predicted protein
T170659c2g1i1	434	T	A	LG20	55,172,526	0.34	0.19	0.40		Low-density lipoprotein receptor-related protein 6 |hypothetical weakly similar
T171446c2g1i3	159	C	G			0.48	0.15	0.55		
T174484c7g1i1	742	C	T	LG23	12,588,360	0.32	0.23	0.40	Syn	RNA-binding protein Nova-1-like, isoform X1
T174834c7g3i2	629	G	C	LG4p	9,417,520	0.38	0.25	0.60	T to R	TRPM8 channel-associated factor homolog, isoform X2
T175088c0g3i1	698	T	C	LG18	9,241,944	0.33	0.19	0.46	UTR	ADP-ribosylation factor 1 like
T175804c1g1i1	766	C	G	LG31	15,851,598	0.32	0.17	0.31	R to G	protein FAM46A-like
T193474c0g1i1	1331	G	A	LG14	38,002,543	0.33	0.19	0.46	Syn	Cytochrome c-type heme lyase
**Private to PL**										
T44119c0g1i1	152	A	C	LG17	8,115,846	0.47	0.31	0.48		Glutamine–tRNA ligase
T70707c0g1i1	1085	T	C	LG14	14,039,993	0.47	0.33	0.48	UTR	Costars family protein ABRACL
T70707c0g1i1	1506	T	C	LG14	14,040,414	0.43	0.31	0.45	UTR	Costars family protein ABRACL
T83913c0g1i1	419	G	A	LG18	59,124,877	0.55	0.38	0.67	UTR	Adenosine kinase
T83913c0g1i1*	1075	C	T	LG18	59,122,320	0.66	0.60	0.73	D to N	Adenosine kinase
T106859c0g1i1	366	G	T	LG14	14,052,297	0.46	0.25	0.47		beta-taxilin, isoform X2
T140173c0g1i2	148	C	T	Scf2193	34,736	0.41	0.35	0.38	UTR	N-myc proto-oncogene protein-like, isoform X2
T148137c3g1i2	1158	G	C	Scf16416	190,147	0.45	0.39	0.56	UTR	dolichyldiphosphatase 1-like
T148137c3g1i2	1432;1440	G(N7)T	T(N7)A	Scf16416	189,864	0.44	0.30	0.50	UTR	dolichyldiphosphatase 1-like
T154282c0g1i1	142	C	G	LG17	8,115,335	0.44	0.32	0.48		Glutamine–tRNA ligase
T154282c0g1i1	252;292;304	G(N39)A -(N11)A	A(N39)T -(N11)C	LG17	8,115,195	0.52	0.37	0.58		Glutamine–tRNA ligase
T154282c0g1i1	575	G	T	LG17	8,114,902	0.41	0.28	0.43		Glutamine–tRNA ligase
T156040c0g1i1	747	C	A	LG4q.1:29	23,198,437	0.37	0.22	0.46	UTR	Vacuolar protein sorting-associated protein 29
T159843c0g1i1	1331	G	A	LG31	24,512,078	0.40	0.26	0.39	UTR	chromobox protein homolog 3-like, isoform X1
T159843c0g1i1	1796	G	T	LG31	24,512,546	0.58	0.44	0.55	UTR	chromobox protein homolog 3-like, isoform X1
T161458c2g1i1	77	A	G	LG6.2	13,677,880	0.34	0.22	0.54		cyclin A2, isoform X2
T161984c0g1i1	618	C	T	LG31	22,464,046	0.33	0.20	0.33	Syn	Trafficking protein particle complex subunit 3, isoform X1
T162197c14g1i1	1353	G	T	LG7	16,955,663	0.53	0.39	0.54	Syn	Protein LZIC
T163256c7g7i3	3892	T	A	Scf1703	56,973	0.54	0.40	0.51	UTR	CIGSSA_123112.t11
T163256c7g7i3	4065	A	C	Scf1703	56,800	0.47	0.34	0.56	UTR	CIGSSA_123112.t11
T163989c5g1i1	1293	T	C	LG7	27,364,962	0.36	0.11	0.52	UTR	fibrous sheath CABYR-binding protein-like, isoform X1
T165162c16g1i1	820	G	A	LG1	17,578,711	0.34	0.15	0.26	UTR	TOX high mobility group box family member 2-like, isoform X4
T165162c16g1i1	974	C	T	LG1	17,578,865	0.32	0.14	0.31	UTR	TOX high mobility group box family member 2-like, isoform X4

Continued on Next Page…

Table S3: Variants only found within one morph. Position within contig and genome, alleles, frequency within the morph, F_{ST}-values and predicted effect are shown.

Transcript id	Var pos	Ref	Alt	Chr	Chr pos	Freq	F_{ST}	F_{PT}	Effect	Gene name
T165162c16g1i1	975;984	A(N8)A	T(N8)C	LG1	17,578,870	0.33	0.13	0.38	UTR	TOX high mobility group box family member 2-like, isoform X4
T168092c2g1i5	2724;2726	TTA	ATT	LG7	26,670,845	0.35	0.23	0.51	UTR	45 kDa calcium-binding protein
T168398c0g1i4	1759	G	T	LG32	20,522,655	0.33	0.28	0.35	P to H	Kinesin-like protein
T168651c0g1i1	863	A	G	LG1	17,539,258	0.36	0.30	0.40		alpha-1-syntrophin-like, isoform X1
T168962c1g1i2	994	G	C	LG14	7,560,825	0.35	0.24	0.38	S to T	translocating chain-associated membrane protein 2-like
T169544c12g4i1	442	A	C	LG41	294,246	0.48	0.34	0.53	UTR	CIGSSA_056585.t2
T170480c1g1i1	2202	A	T	LG4q.2	9,848,462	0.34	0.16	0.49		CIGSSA_044851.t3
T170529c17g1i1	176	A	T	LG36	28,810,075	0.40	0.31	0.49		integral membrane protein 2B-like, isoform X1
T171302c3g1i1	521	C	T	LG17	8,116,418	0.42	0.24	0.39		Glutamine–tRNA ligase
T171326c3g1i2	1206	A	T	LG11	21,367,985	0.33	0.15	0.48	UTR	Peptidylarginine deiminase hypothetical
T171686c7g2i1	193	G	C	LG17	8,312,053	0.49	0.39	0.50		Zgc:110709
T171686c7g2i1	665	G	C	LG17	8,311,589	0.53	0.42	0.58		Zgc:110709
T171686c7g2i1	982	T	A	LG17	8,311,272	0.50	0.43	0.50		Zgc:110709
T171686c7g2i1	1424	T	G	LG17	8,310,831	0.42	0.33	0.46		Zgc:110709
T172162c0g1i1	425	T	A	LG7	16,736,407	0.47	0.35	0.54		TAR DNA binding protein
T172217c4g4i1	1238	T	A	LG32	6,053,989	0.53	0.36	0.57	UTR	WW domain-containing adapter protein with coiled-coil
T172217c4g4i1	2113	A	G	LG32	6,054,862	0.58	0.47	0.58	UTR	WW domain-containing adapter protein with coiled-coil
T172357c4g1i1	2197	A	C			0.42	0.41	0.38	Syn,UTR	coronin-7-like
T172967c3g1i1	199	G	C	LG35	8,330,659	0.51	0.35	0.51		RAB11a member RAS oncogene family
T172992c11g4i1	448	T	C	LG14	14,078,902	0.57	0.44	0.60	Syn	Beta-taxilin|hypothetical
T172933c13g1i1	2089	C	T	LG7	16,959,001	0.48	0.33	0.47		calsyntenin 1, isoform X4
T173769c10g1i2	742;743;748	TATTTTT	ATTTTTG	Scf1321	226,642	0.34	0.18	0.49		Inosine-5-monophosphate dehydrogenase 1
T174499c6g3i3	1150	A	G	LG7	16,917,732	0.50	0.38	0.53	S to G	ubiquitination factor E4B, UFD2 homolog (S. cerevisiae), isoform X1
T174814c3g1i1	411	T	A	LG4q.1:29	37,790,310	0.33	0.24	0.47		CMP-N-acetylneuraminate-poly-alpha-28-sialyltransferase
T174821c6g1i1	967	C	G	LG36	31,081,180	0.41	0.35	0.40		unconventional myosin-Ib-like, isoform X1
T174821c6g1i1	1199	G	A	LG36	31,080,948	0.37	0.23	0.36		unconventional myosin-Ib-like, isoform X1
T175009c8g1i2	2905	C	T	LG30	8,090,556	0.47	0.34	0.45	UTR	ATP-binding cassette sub-family F member 1-like
T175236c1g3i4	5881	T	G	LG31	24,323,360	0.61	0.45	0.56	UTR	collagen alpha-1(XVI) chain-like, isoform X1
T175348c1g10i1	258	A	G	LG20	21,264,718	0.32	0.18	0.40		ubiquitin domain containing 2
T175403c90g7i1	1245	A	C	Scf396	147,836	0.73	0.55	0.83	UTR	peptidyl-prolyl cis-trans isomerase FKBP3-like
T175479c1g2i5	407	A	C	LG35	8,345,621	0.50	0.38	0.50	V to G	Ubiquilin-4
T176050c1g1i1	5019	C	A	LG35	8,316,952	0.52	0.44	0.51	UTR	RNA-binding protein MEX3A
T210338c0g1i1	1772	G	A	LG17	8,125,541	0.50	0.36	0.48	Syn	glutaminyl-tRNA synthetase
**Private to SB**										
T145511c0g1i1	2002	T	C	Scf4114	11,937	0.26	0.26	0.25	Syn	Myelin expression factor 2|hypothetical
T160725c0g1i2	919	A	G	LG17	22,513,650	0.40	0.41	0.37	Syn	transcription factor 15-like
T162598c0g1i1	729;742	G(N12)C	A(N12)T	Scf1046	1,457	0.44	0.23	0.44		chromobox homolog 6
T165516c2g3i4	382	C	A	Scf4168	5,044	0.27	0.19	0.46	UTR	
T165516c2g3i4	873;876;912; 924;927	CCAC(N35)A -(N10)AAAT	TCAT(N35)T -(N10)GAAC	LG18	24,320,993	0.43	0.38	0.65	Syn	
T168835c3g2i2	1142	T	A	LG4q.1:29	21,965,763	0.33	0.33	0.37	UTR	Phosphoglycerate mutase 2
T169028c43g1i1	3476	A	C	LG32	2,199,330	0.31	0.29	0.40	UTR	Muscle-related coiled-coil protein

Continued on Next Page…

Table S3: Variants only found within one morph. Position within contig and genome, alleles, frequency within the morph, F_{ST}-values and predicted effect are shown.

Transcript id	Var pos	Ref	Alt	Chr	Chr pos	Freq	F_{ST}	F_{PT}	Effect	Gene name
T169667c0g1i4	2617	A	G	LG18	12,429,904	0.28	0.30	0.32	F to S	DNA topoisomerase 2-alpha
T169667c0g1i4	2835	G	A	LG18	12,430,772	0.28	0.31	0.32	Syn	DNA topoisomerase 2-alpha
T169667c0g1i4	3054	G	T	LG18	12,431,454	0.28	0.30	0.30	Syn	DNA topoisomerase 2-alpha
T169667c0g1i4	3198	A	G	LG18	12,431,726	0.26	0.30	0.30	Syn	DNA topoisomerase 2-alpha
T169667c0g1i4	3375	A	G	LG18	12,432,251	0.29	0.31	0.31	Syn	DNA topoisomerase 2-alpha
T169667c0g1i4	3447	G	A	LG18	12,432,602	0.29	0.31	0.36	Syn	DNA topoisomerase 2-alpha
T169667c0g1i4	3672	T	C	LG18	12,433,037	0.28	0.32	0.32	Syn	DNA topoisomerase 2-alpha
T169667c0g1i4	4308	C	A	LG18	12,435,579	0.27	0.29	0.32	Syn	DNA topoisomerase 2-alpha
T170294c11g5i4	1744	T	C	LG17	22,247,612	0.33	0.31	0.32	Syn	ras-related protein Rap-1A-like, isoform X1
T173826c6g1i1	342	A	G	LG28	18,758,190	0.36	0.32	0.43		Si:dkey-77p13.2
T210331c21g1i1	4709	G	A	MG	3,411	0.74	0.68	0.79	UTR	

*Validated in KASP-assay

Table S4: Gene ontology categories enriched in variants private to PL-charr ($p < 0.01$ in the other morphs) on the transcripts (tr) and gene (ge) level. The number of transcripts and genes with observed private variants (PL_{tr} and PL_{ge}) and the total number of transcripts and genes tested in the category (Tot_{tr} and Tot_{ge}) are shown. The multiple testing corrected p-value or false discovery rate (FDR) is also shown for both levels.

Category	Term	PL_{tr}	Tot_{tr}	FDR_{tr}	PL_{ge}	Tot_{ge}	FDR_{ge}
GO:0006425	glutaminyl-tRNA aminoacylation	4	4	2.47e-06	1	1	1.0000
GO:0006433	prolyl-tRNA aminoacylation	4	4	2.47e-06	1	1	1.0000
GO:0006424	glutamyl-tRNA aminoacylation	4	6	2.44e-05	1	3	1.0000

Table S5: Genetic polymorphisms studied in the population sample of Arctic charr.

On figshare: doi:10.6084/m9.figshare.8719784

Transcript_ID: Name of the transcript from the Trinity assembly
Variant: Name of variant.
Gene: Short name of gene.
Gene_name: Long name of gene.
Start: Position of polymorphism within contig.
Ref: Base(s) of the reference allele.
Alt: Base(s) of the alternative allele.
Outg: Base(s) in the salmonid outgroup.
Chr_NCBI_id: Sequence identifier for chromosome or scaffold in genome.
Chr: Name of chromosome.
Chr_position: Position mapped to in chromosome.
Effect: Which effect does the mutation have on the gene, 3'- and 5' UTR indicate mutations in those regions, synonymous do not change the reading frame but mutations that do so are coded as transitions from one amino acid to another (single letter a.a. code)
Marker_ID: Abbreviated ID of each marker.
Sequence: The sequence used to design the KASP assay, the polymorphism are marked by e.g. [A/T].

Table S6: Datafile of the genotypes for the 22 markers scored in the population samples from the four sympatric charr morphs (coded by bases, missing data indicated by "NA").

On figshare: doi:10.6084/m9.figshare.8721317

Table S7: Estimates of F-statistics for the entire KASP data and tests of Hardy-Weinberg proportions for the entire KASP dataset (Total) and individual morphs.

Variant	Total (P)	SB (P)	LB (P)	PL (P)	PI (P)	F_{ST}	F_{IS}	F_{IT}
eif4g2b_G652A	0.0	1.0	1.0	0.546	0.400	0.47	0.15	0.55
timp2b_G355A	0.076	1.0	0.039	0.488	1.0	0.10	0.14	0.23
tcf15_C1080T	0.0	1.0	0.515	1.0	0.021	0.42	0.14	0.50
lrrc1_G2814A	0.0	1.0	1.0	1.0	1.0	0.54	0.00	0.54
sox21b_T317G	0.001	1.0	1.0	1.0	0.378	0.43	0.05	0.46
calm1_C1027A	0.0	1.0	0.369	1.0	0.638	0.51	0.02	0.52
tmem9b_C649T	1.0	0.436	0.343	1.0	0.619	0.22	-0.20	0.07
pairb_C2016T	0.122	0.546	1.0	1.0	1.0	0.31	-0.09	0.25
Kiaa1324_TC393AA	0.0	1.0	1.0	1.0	0.026	0.46	0.21	0.58
gas1l_A3641C	0.0	1.0	1.0	1.0	1.0	0.74	-0.12	0.71
gabpb1_G238A	0.0	1.0	1.0	1.0	1.0	0.66	-0.10	0.62
msi1_A1594T	0.002	0.328	0.586	0.373	0.597	0.31	0.10	0.37
cox11_C1202A	0.0	1.0	1.0	1.0	1.0	0.52	-0.01	0.51
gnl3l_G795T	0.013	0.399	1.0	1.0	0.444	0.21	0.14	0.32
eif4ebp2_T1083A	0.410	1.0	1.0	0.291	0.108	0.28	-0.18	0.15
lrp10_A775C	0.0	0.560	1.0	1.0	1.0	0.63	0.03	0.64
neurod1_T1187C	0.001	0.553	0.077	0.426	0.502	0.30	0.15	0.40
dennd5a_A2555T	0.0	1.0	1.0	1.0	1.0	0.69	-0.09	0.66
Sik3_A5236G	0.0	1.0	0.013	1.0	0.091	0.42	0.19	0.53
cdkn2a_A257G	0.149	1.0	1.0	1.0	0.022	0.11	0.09	0.19
wee1_T305A	0.0	1.0	1.0	1.0	1.0	0.72	-0.10	0.69
adk_C1075T	0.0	0.336	1.0	1.0	0.347	0.40	0.18	0.50

Variant: Name of variant
Total (P): Significance of the test for Hardy Weinberg proportions as estimated from Fisher's exact tests, on the entire dataset
SB (P): Significance of the test for Hardy Weinberg proportions as estimated from Fisher's exact tests for SB
LB (P): Significance of the test for Hardy Weinberg proportions as estimated from Fisher's exact tests for LB
PL (P): Significance of the test for Hardy Weinberg proportions as estimated from Fisher's exact tests for PL
PI (P): Significance of the test for Hardy Weinberg proportions as estimated from Fisher's exact tests for PI
F_{ST} : F-statistics for variation between morphs
F_{IS} : F-statistics for variation within population
F_{IT} : F-statistics for variation between individuals

Table S8: Datafile of the LD (r^2) for all pairs of markers, by morph

On figshare: doi:10.6084/m9.figshare.8721455

Supplementary Figures

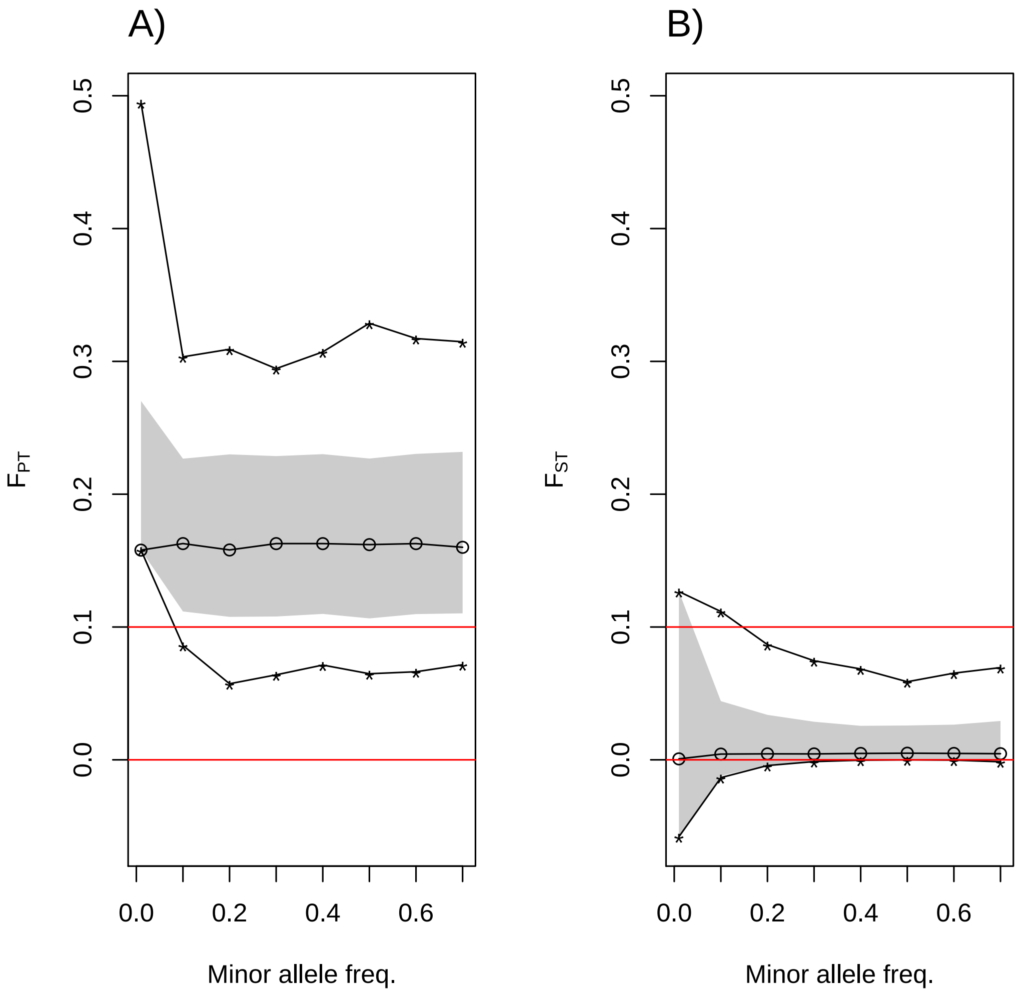

Figure S1: Results from simulations on F-statistics for a biallelic variant with the same allele frequency among morphs. The gray area indicate the 95% confidence area. The median, minimum and maximum values are shown by dots and lines. A) Shows F_{PT} values and B) F_{ST} values. As expected F_{PT} values are high and we chose 0.1 (red horizontal line) as cutoff as it is outside the 95% confidence area in the simulations.

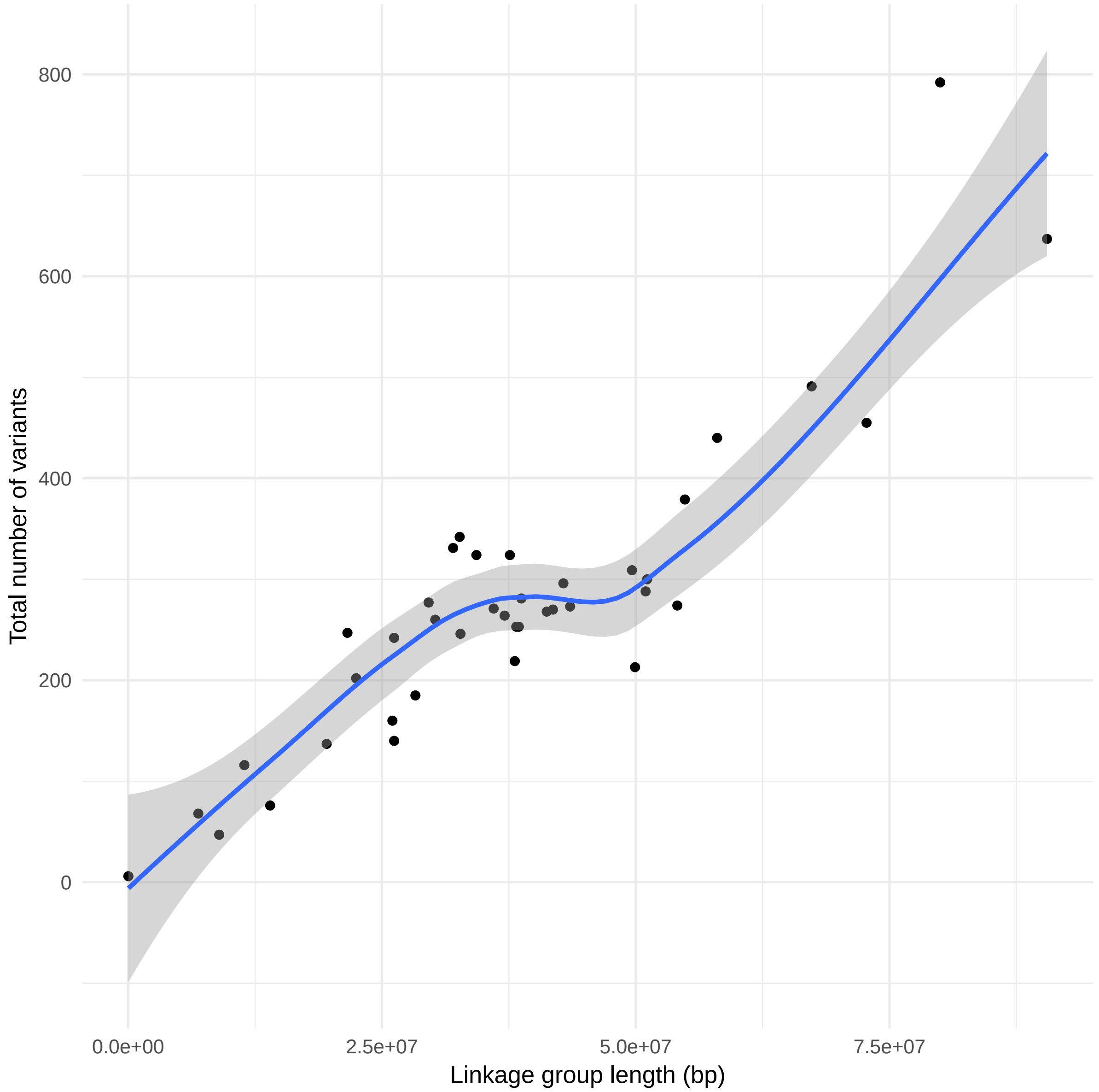

Figure S2: Total number of transcriptome variants by linkage group length. A loess smooth curve (blue) with 95% confident interval (gray) is also graphed.

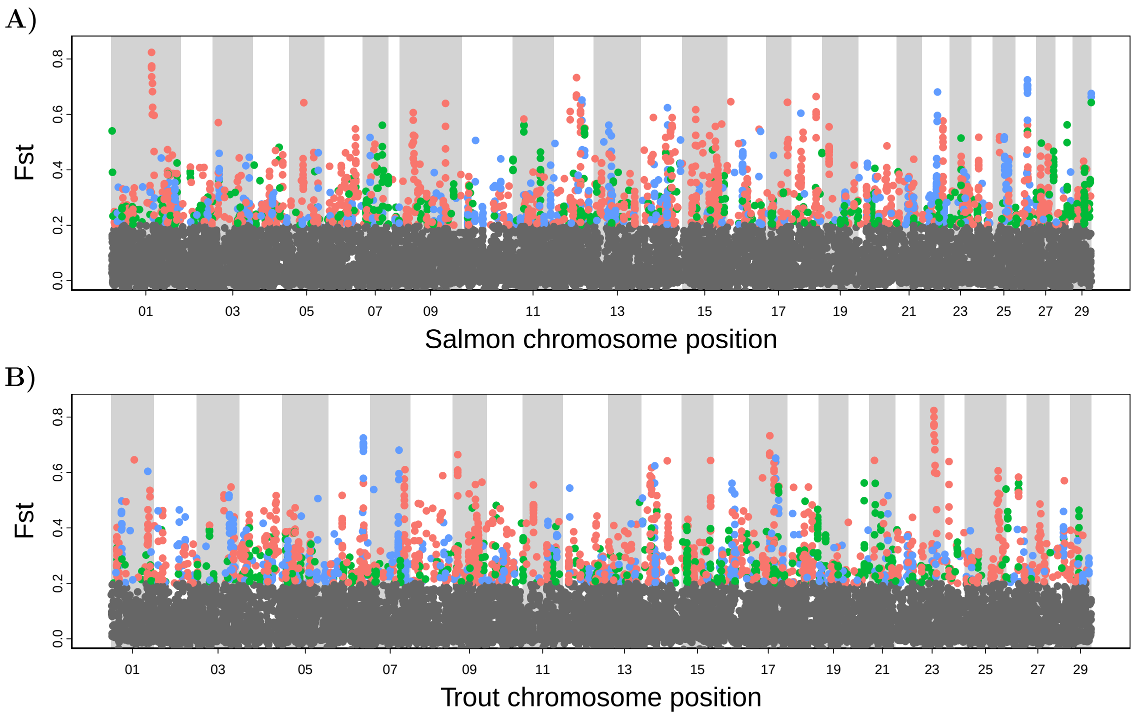

Figure S3: F_{ST} values plotted by position of variants on the salmon **A)** and rainbow trout **B)** genome. The colors indicate which morphs differs most strongly in allele frequency from the other two for variants with $F_{ST} > 0.2$. Red PL, blue SB and green LB

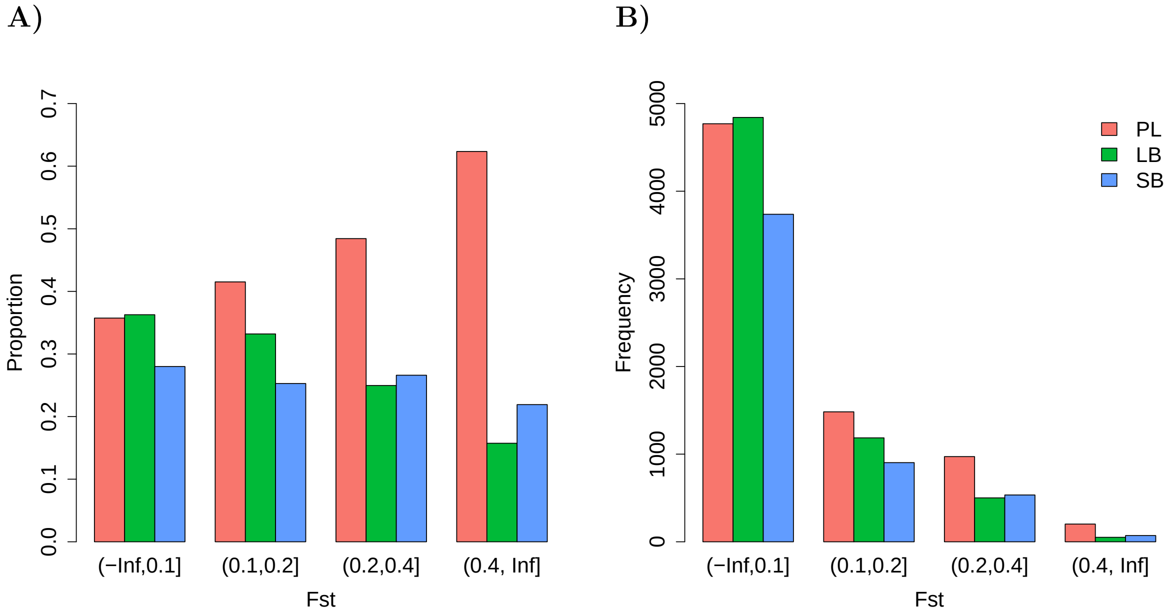

Figure S4: **A)** Proportion of variants (within F_{ST} groups) for different F_{ST}-values grouped by the morph with highest deviation in allele frequency from the other two. **B)** The number of variants in each F_{ST} category grouped by the morph with highest deviation in allele frequency from the other two. The legend in **B)** also applies to **A)**.

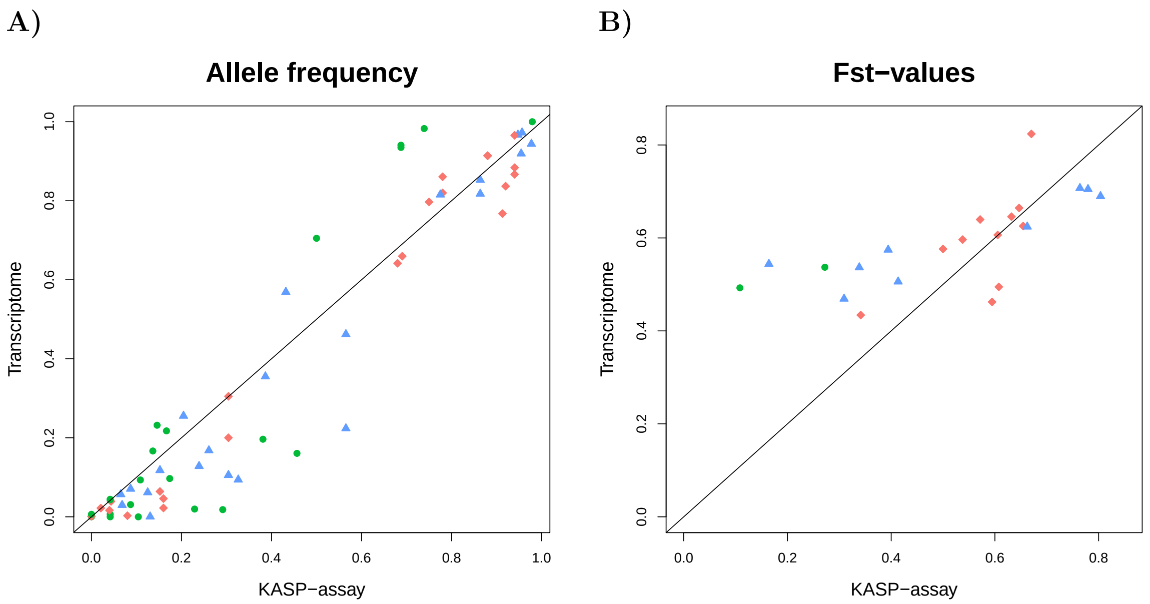

Figure S5: Tight relationship was found between the allele frequencies and F_{ST} values estimated from the transcriptome and the population genetic sample (Kasp assay). **A)** The allele frequencies for the 22 markers were estimated for each morph (color coded) and the estimates from the two methods show high positive correlation (Kendall's $\tau = 0.76$, Pearson's $r = 0.96$, $p < 0.0001$). The diagonal line represents the 1:1 relationship. **B)** F_{ST}-values calculated for the transcriptome and the population sample had comparatively weaker association (Kendall's $\tau = 0.58$, Pearsons's $r = 0.70$, $p < 0.001$). Notably, two markers deviated substantially between the two methods (in *timp2b* and *cdkn2a*), with higher F_{ST} in the transcriptome compared to the population sample (due to underestimation of some rarer allele frequencies in the transcriptome). The colors indicate which morphs shows the highest deviation from the other two in mean allele frequency for each marker in the transcriptome (SB: blue, LB: green and PL: red).

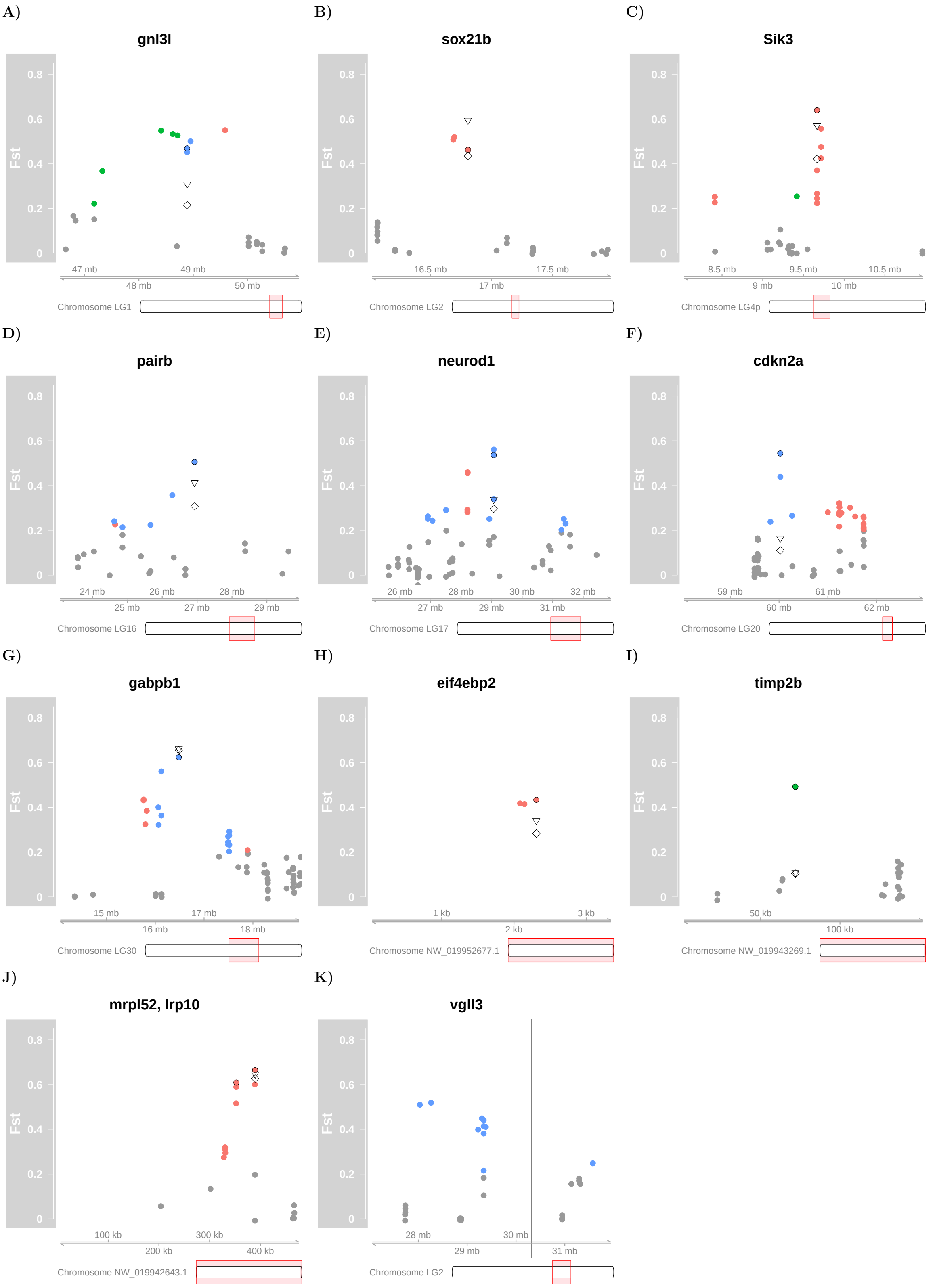

Figure S6: **A-J)** Detailed view of chromosomal regions of variants in KASP-assay and nearby transcriptome variants (not-validated). The colored dots mark F_{ST} values from the transcriptome as in Fig. 3 and the variants taken for validation are marked with a filled black circl (○). The triangles (▽) show the F_{ST} value from the KASP-assay for the three transcriptome morphs (PL, SB and LB) and the diamonds (◇) F_{ST} for all morphs (including PI). **K)** Detailed view of variants nearby the *vgll3* locus. The vertical line represents the location of the *vgll3* gene, not transcribed and thus no variants detected, using the same color code (SB: blue, LB: green and PL: red) for variants.
